# Pregnancy Loss and Iodine Status: The LIFE Prospective Cohort Study

**DOI:** 10.3390/nu11030534

**Published:** 2019-03-01

**Authors:** James L. Mills, Mehnaz Ali, Germaine M. Buck Louis, Kurunthachalam Kannan, Jennifer Weck, Yanjian Wan, Joe Maisog, Andreas Giannakou, Rajeshwari Sundaram

**Affiliations:** 1 Epidemiology Branch, Division of Intramural Population Health Research, Eunice Kennedy Shriver National Institute of Child Health and Human Development, National Institutes of Health, Bethesda, MD 20892, USA; mehnaz.ali93@gmail.com (M.A.); andersgian@gmail.com (A.G.); 2 Office of the Director, Division of Intramural Population Health Research, Eunice Kennedy Shriver National Institute of Child Health and Human Development, National Institutes of Health, Bethesda, MD 20892, USA; glouis@gmu.edu (G.M.B.L.); jennifer.weck@nih.gov (J.W.); 3 Dean’s Office, College of Health and Human Services, George Mason University, Fairfax, VA 22030, USA; 4 Wadsworth Center, New York State Department of Health, Albany, NY 12201, USA; kurunthachalam.kannan@health.ny.gov (K.K.); Yanjian.Wan@whcdc.org (Y.W.); 5 Glotech, Inc., Rockville, MD 20850, USA; bravas02@gmail.com; 6 Biostatistics and Bioinformatics Branch, Division of Intramural Population Health Research, Eunice Kennedy Shriver National Institute of Child Health and Human Development, National Institutes of Health, Bethesda, MD 20892, USA; sundaramr2@mail.nih.gov

**Keywords:** iodine, pregnancy loss, spontaneous abortion, miscarriage, fetal loss

## Abstract

Iodine deficiency in pregnancy is a common problem in the United States and parts of Europe, but whether iodine deficiency is associated with increased pregnancy loss has not been well studied. The LIFE study provided an excellent opportunity to examine the relationship between iodine status and pregnancy loss because women were monitored prospectively to ensure excellent ascertainment of conceptions. The LIFE study, a population-based prospective cohort study, monitored 501 women who had discontinued contraception within two months to become pregnant; 329 became pregnant, had urinary iodine concentrations measured on samples collected at enrollment, and were followed up to determine pregnancy outcomes. Of the 329, 196 had live births (59.5%), 92 (28.0%) had losses, and 41 (12.5%) withdrew or were lost to follow up. Urinary iodine concentrations were in the deficiency range in 59.6% of the participants. The risk of loss, however, was not elevated in the mildly deficient group (hazard ratio 0.69, 95% confidence interval 0.34, 1.38), the moderately deficient group (hazard ratio 0.81, 95% confidence interval 0.43, 1.51), or the severely deficient group (hazard ratio 0.69, 95% confidence interval 0.32, 1.50). Iodine deficiency, even when moderate to severe, was not associated with increased rates of pregnancy loss. This study provides some reassurance that iodine deficiency at levels seen in many developed countries does not increase the risk of pregnancy loss.

## 1. Introduction

Inadequate maternal iodine stores during pregnancy result in insufficient thyroid hormone production with serious adverse effects in the offspring. In areas where there is severe iodine deficiency, congenital hypothyroidism causes goiter, growth retardation, and neurological damage including intellectual disability. Thus, ensuring adequate iodine intake is critical for normal fetal development [1]. 

Thyroid deficiency has been reported to increase the risk for pregnancy loss as well as abnormal fetal development; however, whether iodine status is related to fetal loss is less clear [2]. This is an important problem because US pregnant women had a median urinary iodine concentration of 129 μg/L [3], below the range of 150–249 μg/L recommended by the World Health Organization (WHO) [4]. In Europe, urinary iodine excretion in pregnant women in eight iodine deficient countries was between 35 and 150 μg/L, most well below the range defined by the WHO as sufficient [5]. 

Few studies have examined the relationship between iodine and pregnancy loss in the past, and the information on early losses is very limited. Some studies have focused on stillbirths and postnatal losses due to congenital malformations, others have not actually measured iodine, and still others were ecological studies comparing different geographical areas [6,7,8,9,10]. To our knowledge, no prospective cohort studies to date have recruited women prior to conception, identified pregnancies by early human chorionic gonadotropin measurement and identified losses from very early pregnancy. In fact, few studies have examined losses before the third trimester of pregnancy.

The high prevalence of mild to moderate iodine deficiency in pregnant women is an important reason to determine whether iodine contributes to early pregnancy losses. Our previous study showed that women with poor iodine status had reduced fecundability [11]. This investigation was conducted to determine whether low urinary iodine concentration is associated with an increase in pregnancy losses. We hypothesized that the pregnancy loss rate would be higher in the group of women whose urinary iodine concentration was below the WHO sufficiency range than in the group whose urinary iodine concentration was within the sufficiency range.

## 2. Materials and Methods 

### 2.1. Study Population

The LIFE Study recruited couples who had discontinued contraceptive use in the last 2 months for the purpose of becoming pregnant between 2005 and 2009 from 16 counties in Michigan and Texas. The study used Texas hunting and fishing licenses and a Michigan commercial marketing database to provide a defined, although not representative, population of couples planning pregnancy [12].

In order to be eligible to enroll in the study women had to be in a committed relationship; be able to communicate in English or Spanish; be aged 18–40 with a partner 18 years or older; have menstrual cycles between 21 and 42 days; have no history of injectable hormonal contraception in the past year or breastfeeding in the last six months; have no clinically diagnosed infertility in either partner; and be off contraception for less than 2 months. Prior to enrollment, women’s urine was tested to ensure they were not pregnant. 

Ethical approval: Full human subjects approval was obtained from all participating institutions and all couples gave informed consent before any data collection.

Institutional Review Board Approvals: RTI #8949, Texas A & M University #2004, The EMMES Corporation #31411, CDC #4489, Wadsworth Center, NYS Department of Health #11-011, NIH OHRP Assurance FWA # 00005897.

### 2.2. Study Protocol

Following enrollment, couples were interviewed individually to obtain information on factors potentially affecting pregnancy. Measurements of height and weight were taken at the time of the interview to calculate body mass index (BMI). Couples were instructed to keep daily journals on relevant lifestyle information such as smoking and consumption of alcoholic and caffeinated beverages. Women reported on sexual intercourse, menstruation, and home pregnancy test results. The journals were kept until a positive home pregnancy test or after 12 months of trying. Pregnant women continued to keep journals through seven post-conception weeks of gestation, followed by monthly journals until delivery or a loss. 

Women were trained to use the Clearblue™ fertility monitor, which tracks estrone-3-glucuronide and luteinizing hormone, to predict the day of ovulation in order for couples to time intercourse to optimize their chance of conceiving. They were also trained in the use of the Clearblue™ digital pregnancy test which can identify pregnancies through detection of 25 mIU/mL of human chorionic gonadotropin (hCG) [13]. Pregnancy was identified by a positive hCG test on the expected day of menstruation. Women were asked to record the results of their tests in a daily journal. Pregnancy loss was identified by conversion to a negative hCG test, clinical confirmation, or onset of menstruation depending on gestational dating. 

Spot urine samples used to measure iodine and creatinine were collected at the first in-home interview. At enrollment, serum samples were collected for cotinine. We were unable to measure thyroid hormones. 

### 2.3. Statistical Methods

We first characterized the cohort by pregnancy outcome to identify factors associated with loss, with significance based on either the χ^2^ or the Kruskal–Wallis test. Descriptive characteristics of the study population were compared between categories of iodine concentrations, namely normal, mild, moderate, and severe deficiency (as defined below), based on whether a participant had a successful pregnancy, experienced a loss, or was lost to follow-up. Differences were assessed using the Student’s *t*-test or Wilcoxon nonparametric test for continuous data and chi-squared test or Fisher’s exact test for categorical data where appropriate. 

We defined time to pregnancy loss as the number of observed days between ovulation or the monitor’s peak fertility day (LH surge) and the date of the reported loss. The peak (LH) monitor day was assumed to be the day of conception, given the ovum’s short (≈24 h) interval for fertilization. We estimated the distribution of time to pregnancy loss after conception using survival analysis techniques, while accounting for right censoring (withdrawals/births) of the gestational age (in days) from the time of ovulation. 

We performed the analysis with respect to iodine in two ways: iodine concentrations and iodine creatinine ratios. Iodine concentrations were classified based on World Health Organization [4] cut-offs: normal (150 or greater µg/L), mild deficiency (100–149 µg/L), moderate deficiency (50–99 µg/L) and severe deficiency (less than 50 µg/L). We categorized iodine creatinine ratios as below 50 µg/g, 50 to 99 µg/g, 100 to 149 µg/g, and 150 µg/g and above. The distribution of iodine concentrations was compared by pregnancy status (pregnant, not pregnant, withdrew) using the Kruskal–Wallis test. 

Cox’s proportional hazards models were fitted on time to loss for iodine defined as described above, providing hazard ratios for estimating the associations of covariates with risk for loss. A hazard ratio less than 1 indicates a reduced risk for loss. We adjusted for confounders chosen *a priori* based on the literature [14]: woman’s age, difference between female and male partner’s age, woman’s race/ethnicity (referent: non-Hispanic white), woman’s educational level (referent: high school or less), household income (referent: less than $29,999), body mass index (normal: less than 25, overweight: 25–29.9 or obese: 30 or greater), diabetes mellitus, periconceptional consumption of alcohol per day, periconceptional smoking per day (categorized as non-smokers (cotinine <3 ng/mL), passive (cotinine 3 ng/mL to <10 ng/mL), and active (at least 10 ng/mL) [15]), periconceptional consumption of caffeine per day, previous losses conditional on getting pregnant or not (referent: not pregnant), history of hypothyroid disease (referent: no), history of hyperthyroid disease (referent: no) and creatinine (log-transformed).

We performed sensitivity analyses to determine the robustness of our primary analysis by repeating the analysis excluding (i) all women with a history of hypothyroidism or hyperthyroidism, (ii) all women with a history of hypothyroidism, (iii) women being treated for hypothyroidism or hyperthyroidism, and (iv) women being treated for hypothyroidism. We also performed the analysis of iodine (log-transformed) as a continuous exposure. Additionally, all the above-mentioned analyses were performed on iodine to creatinine ratios with iodine in categories and as a continuous measure. We assessed the functional form of iodine as well as the iodine-to-creatinine ratio using splines. This was achieved by fitting a cubic spline with five knots placed at equally spaced percentiles, including minimum and maximum values, on log-transformed iodine concentrations.

### 2.4. Sensitivity Analysis

We also did sensitivity analysis to account for the potential selection bias of not including couples who did not get pregnant and may have had lower iodine levels. This was achieved by fitting logistic regression to estimate and assign inverse probability censoring weights (IPCW) of achieving an observed singleton pregnancy accounting for those couples not becoming pregnant. To determine the probability that we could have missed a true effect of low iodine status on losses, we estimated the probability of a relative risk of at least “*r*”, e.g., *r* = 2 for a relative risk of two or more for each iodine deficient group with respect to the sufficient group based on our estimated Cox model. All of the analyses were implemented in SAS for Windows version 9.4, Cary, NC, USA.

### 2.5. Laboratory Methods

Each urine sample (100 μL) was mixed with 1 µL of acetic acid and 1 μL of ascorbic acid [16,17] and incubated at room temperature for 10 min. After tetramethylammonium hydroxide (TMAH) digestion was performed at 90 °C [18,19], 1.87 mL of water and 1.5 μL of acetic acid were added and centrifuged for 15 min at 4000× *g*. The supernatant was injected into HPLC-MS/MS (Quattro LC, Micromass, Waters Corporation, Milford, MA, USA) with a 250 mm × 2 mm IonPac AS-21 anion exchange column (Dionex, Sunnyvale, CA, USA). An isocratic mobile phase of 20 mM aqueous methylamine was used at a flow rate of 300 µL/min. Iodide was monitored by the mass transition of m/z 127→m/z 127 for I-. The cone voltage and the collision energy were 40 V and 22 V, respectively. The limit of quantitation for urinary iodide was 5 ng/mL. and the detection frequency was 100%, with the range of 6.2 ng/mL to 1664 ng/mL. Duplicate and matrix spike samples were included in each batch of 50–75 samples analyzed. Iodide was not detected in procedural blanks. Reference urine samples from the Centers for Disease Control and Prevention of known iodide concentration (from EQUIP, Ensuring the Quality of Iodine Procedures) were also analyzed with each batch of samples. Laboratory personnel were masked to all other study data. 

## 3. Results

Of the 501 couples enrolled in the study, 347 (69.3%) became pregnant. Three twin or ectopic pregnancies were excluded. Iodine could be measured in 329 (95.6%) of the remaining 344 women; 196 resulted in live births (59.5%), 92 (28.0%) resulted in losses, and 41 (12.5%) withdrew or were lost to follow up. 

Table 1 lists the characteristics of women who had pregnancies resulting in live births, losses, or who left the study prior to a loss or live birth. Women who did not complete the study were significantly younger (*p* = 0.006) and less likely to consume alcohol (*p* = 0.04) than those who completed the study. Women who had live births and women who experienced losses did not differ significantly in age, race, education, household income, BMI, parity, previous losses, smoking, alcohol consumption, or caffeine intake. There were 24 women who had a history of hypothyroidism of whom 20 were on treatment; 3 had a history of hyperthyroidism of whom 2 were receiving treatment.

Only 133 women (40.4%) had samples in the iodine sufficient range (150 or greater µg/L); 52 women’s samples (15.8%) were in the mildly deficient range (100–149 µg/L); 74 (22.5%) were in the moderately deficient range (50–99 µg/L); and 70 (21.3%) were in the severely deficient range (less than 50 µg/L). Age, income, BMI, parity, previous losses, alcohol consumption, and caffeine consumption did not differ significantly by iodine concentration. Modest differences were present by education and race/ethnicity. (Table 2) The mean iodine concentration in the group that withdrew from the study (154.0 µg/L) was not significantly different (*p* = 0.34) from the concentration in the group that experienced a loss (178.6 µg/L) or the group that delivered a live infant (174.6 µg/L).

The group of women whose iodine results were in the deficiency range did not have a significantly different loss rate than those who were in the iodine sufficient group. Table 3 displays the hazard ratio for pregnancy loss by iodine status with the group of iodine sufficient women as a reference. A hazard ratio less than 1 indicates a reduced risk for loss. The hazard ratio for pregnancy loss in the mild iodine deficiency group was 0.65 (95% CI 0.34, 1.24), while the hazard ratio for pregnancy loss in the moderate deficiency group was 0.82 (95% CI 0.48, 1.39) and the hazard ratio for pregnancy loss in the severely deficient group was 0.66 (95% CI 0.37, 1.18). The log iodine hazard ratio for a 1-unit increase in log-transformed iodine for pregnancy loss was 1.11 (95% CI 0.89, 1.38). The hazard ratio for pregnancy loss using the iodine/creatinine ratio for women in the mild iodine deficiency group was 0.85 (95% CI 0.48, 1.50), for women in the moderate deficiency group was 0.77 (95% CI 0.43, 1.39) and for women in the severe deficiency group was 0.58 (95% CI 0.18, 1.85). The log iodine/creatinine hazard ratio was 1.04 (95% CI 0.80, 1.36).

After adjustment the hazard ratio for pregnancy loss in the mild iodine deficiency group was 0.69 (95% CI 0.34, 1.38), in the moderate deficiency group was 0.81 (95% CI 0.43, 1.51) and in the severe deficiency group was 0.69 (95% CI 0.32, 1.50). The log iodine hazard ratio was 1.10 (95% CI 0.81, 1.49). The adjusted hazard ratio for pregnancy loss using the iodine/creatinine ratio in women in the mild iodine deficiency group was 0.72 (95% CI 0.38, 1.36), in the moderate deficiency group was 0.76 (95% CI 0.41, 1.40) and in the severe deficiency group was 0.53 (95% CI 0.16, 1.76). The log iodine/creatinine hazard ratio was 1.05 (95% CI 0.80, 1.38). Thus, there was no significant difference in loss rates by iodine status in any group. 

Sensitivity analyses, excluding all women with a history of hypo- or hyperthyroidism and women being treated for hypo- or hyperthyroidism, did not materially affect the results (Table 3). Testing the functional form of the iodine relationship with losses by splines showed no significant non-linearity in the relationship (see supplementary figure). As noted above, using iodine/creatinine ratios rather than adjusting for creatinine produced very similar results. Our weighted analysis to account for dropouts in the sample due to couples not getting pregnant also did not change the results significantly.

## 4. Discussion

In this first prospective cohort study, women in the iodine deficient groups did not experience a significantly increased rate of losses compared to the group whose samples were in the iodine sufficient range. This is reassuring because of the 329 iodine measurements available, 21.3% were in the severe deficiency range, 22.5% were in the moderate deficiency range and an additional 15.8% were in the mild deficiency range. Given that 55.8% of pregnant women in the USA NHANES study from 2007 to 2010 had iodine deficiency [20], our results provide important reassurance that poor iodine status is not increasing the risk of pregnancy loss. Based on our previous finding that iodine deficiency was associated with reduced fecundity in this population [11], it is possible that women who succeed in becoming pregnancy despite poor iodine status are able to compensate and have successful pregnancies.

To our knowledge, this is the first study to examine iodine status as a risk factor for early pregnancy loss. Although iodine deficiency is cited as a risk factor for early losses based on a WHO report [8], the studies cited by WHO did not actually investigate early pregnancy losses but stillbirths and childhood deaths [9,10,21]. Moreover, tracing the evidence back from the WHO report reveals that many of the investigations available to them had serious limitations for examining losses [7]. A review by McMichael and colleagues relied on ecological studies, which compared countries with and without iodization programs that used vital records data on infant deaths and stillbirth rates [7]. The McMichael study compared stillbirth rates in countries before and after iodization. Although rates decreased over time, no significant difference in rates between countries that did, or did not, begin iodination were found. Ecological studies cannot identify specific causes for adverse events, making it difficult to draw conclusions from these data alone [7]. Because the Pharoah study [21] cited by McMichael and colleagues lumped stillbirths together with infant deaths, it is even more difficult to say whether their results are applicable to losses. Other problems in the cited studies include using serum thyroid hormone levels as a surrogate for actual iodine levels; taking samples from some women up to 4 years after pregnancy [9]; and studying women in an isolated region like Papua New Guinea who may also have other medical problems that affect losses. Yet another study cited examined stillbirths and deaths due to congenital anomalies as their outcomes [10] and thus provides little information on losses.

The studies that included earlier losses have produced mixed results. Jiskra et al. studied losses occurring before the 12th gestational week but did not specify when women entered the study [22]. They found that urinary iodine concentrations were significantly lower in women who had losses than in non-pregnant control women. However, iodine concentrations would be expected to fall during pregnancy because of the demands of the fetus and increased renal excretion; moreover, potential control women were dropped for thyroid disease, raising the possibility that those with lower iodine status would not have been included. Dillon and Milliez collected obstetrical history data by interview and measured urinary iodine in women, only 9% of whom were pregnant at the time, to examine the relationship between iodine deficiency and reproductive failures defined as at least three miscarriages or at least one stillbirth [23]. Women with very severe iodine deficiency were more than three times as likely to report reproductive failures as women who were not deficient—odds ratio of 3.64 (95% CI 2.92, 4.55). Other factors clearly played an important role in this underweight, malnourished population. Underweight women had an odds ratio of 4.60 (95% CI 3.16, 6.71) for reproductive failure and illiterate women had an odds ratio of 8.01 (95% CI 6.31, 10.18) [23]. 

In contrast, Yang and coworkers found no significant difference between iodine status and miscarriage or stillbirth; however, the average gestational age at entry was 27 weeks and women with thyroid disease were excluded [24]. Xiao et al. found a non-significant higher rate of abortions prior to 28 weeks identified by telephone follow up in women in the urinary iodine concentration <100 µg/L group [25]. In the large Avon Longitudinal Study of Parents and Children (ALSPAC) cohort, [26] the authors found no association between iodine status and losses but pointed out that with only 11 prenatal losses identified, further research was required. 

It is worth noting that a number of other studies have investigated the effect of subclinical hypothyroidism on early pregnancy losses, but not did not determine whether iodine deficiency was a factor [27,28,29]. 

Our study was conducted in a developed country and therefore relatively few women had potentially confounding nutritional problems such as those reported in the Dillon paper. Our data provide information on the effects of all levels of iodine deficiency on pregnancy loss including severe iodine deficiency and are more relevant to the iodine status of most women in developed countries. 

This study has several strengths. We believe our study is unique in measuring iodine prior to conception and identifying even very early losses. The majority of pregnancy losses occur very early in pregnancy [30], so most other studies miss a large number of losses by not having women under observation early in gestation. This study uses actual urine iodine measures from time of entry, avoiding the problem that dietary reports of iodine intake are not accurate [31]. Daily journals begun before pregnancy were kept for seven weeks post-conception followed by monthly journals focused on events occurring in specific weeks of gestation for more complete collection of data relating to the pregnancy. Those who experienced losses completed a pregnancy loss card with information on the temporal ordering of signs and symptoms associated with the loss to more accurately pinpoint the time of loss. 

There are several limitations as well. Iodine concentrations are volatile and samples taken from a given woman may vary considerably over time [32], so data must be interpreted by group as we have done, not by individual. Iodine was measured when women entered but excretion might have changed between entry and pregnancy although 90 percent of pregnancies occurred in the first six cycles and most losses occur very early in pregnancy. Nonetheless, some women could have begun iodine containing prenatal supplements between conception and the loss. There were a number of withdrawals during the study; however, we were able to characterize those who withdrew. We showed that there was no bias regarding iodine concentrations in those who withdrew, i.e., their iodine concentrations did not differ significantly from those who completed the study, and we showed how those who withdrew differed from those who remained on risk factors for pregnancy loss. The loss rate in our study (28%) was similar to the best estimate (31%) for studies that identified very early losses [30]. It is a major challenge to recruit women without fertility problems prior to conception. Thus, recruiting 501 such women is an achievement. Nonetheless, our study population is small given that there are many causes of pregnancy loss. Therefore, other studies should be done to confirm our findings. 

We performed a statistical analysis to test the probability that we might have missed a true association between low iodine status and losses. For our sample, the probability that we could have missed a true doubling of risk (RR = 2) was very low, *p* ≤ 0.005. The probability that we would have missed even a much smaller effect was *p* < 0.05 for RR of 1.3, 1.5 and 1.4 in the severe, moderate and mild groups, respectively. Thus, it is unlikely that a clinically important deleterious effect of low iodine status on losses was present in our population.

It is important to note that iodine requirements increase dramatically when women become pregnant [33] so those with deficient excretion levels prior to pregnancy will likely have deficient status during pregnancy as well. The substantial proportion of samples in the deficiency range indicates our population as a whole has suboptimal iodine status. This provides useful confirmatory information consistent with other U.S. data [20] showing that many women enter pregnancy with iodine concentrations below the recommended range.

In summary, this first cohort study of iodine and early pregnancy loss with excellent ascertainment of losses found encouraging results. Although over half of the women had iodine samples in the deficiency range, they did not experience an increased loss rate. This study provides some reassurance that iodine deficiency at levels seen in many developed countries does not increase the risk of pregnancy loss.

## Figures and Tables

**Table 1:** Characteristics of women becoming pregnant by study outcome (*n* = 329).

Variable ^a^	Live Births (*n* = 196)	Losses (*n* = 92)	Withdrew/Lost to Follow-up (*n* = 41)	Total (*n* = 329)	*p*-Value	
					Live Birth Vs. Loss	Live Birth + loss vs. Withdrew/Lost to Follow up
Age (years) *n* (%)					0.06	0.006
29 or less	102 (52.0%)	46 (50.0%)	30 (73.2%)	178 (54.1%)		
30–34	78 (39.8%)	30 (32.6%)	5 (12.2%)	113 (34.3%)		
35 or more	16 (8.2%)	16 (17.4%)	6 (14.6%)	38 (11.6%)		
Race/ethnicity *n* (%)					0.70	0.60
Non-Hispanic white	161 (83.0%)	77 (84.6%)	37 (90.2%)	275 (84.4%)		
Non-Hispanic black	3 (1.5%)	3 (3.3%)	0 (0%)	6 (1.8%)		
Hispanic	17 (8.8%)	6 (6.6%)	3 (7.3%)	26 (8.0%)		
Other	13 (6.7%)	5 (5.5%)	1 (2.4%)	19 (5.8%)		
Education *n* (%)					0.65	0.06
High school or less	8 (4.1%)	6 (6.6%)	0 (0%)	14 (4.3%)		
Some college or technical school	26 (13.4%)	11 (12.1%)	10 (25.0%)	47 (14.5%)		
College graduate or higher	160 (82.5%)	74 (81.3%)	30 (75.0%)	264 (81.2%)		
Household income *n* (%)					0.16	0.67
Less than $29,999	3 (1.6%)	5 (5.6%)	1 (2.4%)	9 (2.8%)		
$30,000–$49,999	23 (12.0%)	6 (6.7%)	3 (7.3%)	32 (10.0%)		
$50,000–$69,999	24 (12.6%)	12 (13.5%)	8 (19.5%)	44 (13.7%)		
At least $70,000	3 (1.6%)	5 (5.6%)	1 (2.4%)	9 (2.8%)		
Body mass index *n* (%)					0.59	0.79
Less than 20	4 (2.0%)	2 (2.2%)	0 (0%)	6 (1.8%)		
20 to 24.9	97 (49.5%)	41 (44.6%)	21 (51.2%)	159 (48.3%)		
25 to 29.9	52 (26.5%)	22 (23.9%)	11 (26.8%)	85 (25.8%)		
30 or greater	43 (21.9%)	27 (29.3%)	9 (22.0%)	79 (24.0%)		
Previous pregnancy losses *n* (%)					0.94	0.47
Never pregnant	77 (39.3%)	36 (39.6%)	15 (37.5%)	128 (39.1%)		
At least 1 loss	13 (6.6%)	7 (7.7%)	5 (12.5%)	25 (7.6%)		
Prior pregnancy, no previous losses	106 (54.1%)	48 (52.7%)	20 (50.0%)	174 (53.2%)		
Smoking *n* (%)					0.43	0.56
None	182 (92.9%)	85 (92.4%)	39 (95.1%)	306 (93.0%)		
Moderate (at least one episode of 1–9 cigarettes per day)	10 (5.1%)	3 (3.3%)	2 (4.9%)	15 (4.6%)		
Heavy (at least one episode of 10 or more cigarettes per day)	4 (2.0%)	4 (4.3%)	0 (0%)	8 (2.4%)		
Alcohol consumption *n* (%)					0.35	0.04
Non-drinker	61 (31.1%)	27 (29.3%)	17 (42.5%)	105 (32.0%)		
Occasional drinker (1–16 drinks per month)	100 (51.0%)	54 (58.7%)	13 (32.5%)	167 (50.9%)		
Drinker (17 or more drinks per month)	35 (17.9%)	11 (12.0%)	10 (25.0%)	56 (17.1%)		
Caffeine intake *n* (%)					0.09	0.31
None	84 (42.9%)	34 (37.0%)	17 (42.5%)	135 (41.2%)		
Moderate (1 cup per day)	75 (38.3%)	30 (32.6%)	18 (45.0%)	123 (37.5%)		
Heavy (2 or more cups per day)	37 (18.9%)	28 (30.4%)	5 (12.5%)	70 (21.3%)		

^a^ Missing values were excluded.

**Table 2:** Medical and socio-demographic factors of women becoming pregnant by World Health Organization categories of urinary iodine concentration (µg/L) (*n* = 329).

Characteristic	Sufficient (≥150 µg/L) (*n* = 133)	Mild Deficiency (100–149 µg/L) (*n* = 52)	Moderate Deficiency (50–99 µg/L) (*n* = 74)	Severe Deficiency (<50 µg/L) (*n* = 70)	Total (*n* = 329)	*p*-Value
Age (Years) *n* (%)						0.09
29 or less	82 (61.7%)	24 (46.2%)	38 (51.4%)	34 (48.6%)	178 (54.1%)	
30–34	37 (27.8%)	24 (46.2%)	29 (39.2%)	23 (32.9%)	113 (34.3%)	
35 or more	14 (10.5%)	4 (7.7%)	7 (9.5%)	13 (18.6%)	38 (11.6%)	
Race/Ethnicity *n* (%)						0.05
Non-Hispanic White	109 (83.2%)	38 (73.1%)	63 (85.1%)	65 (94.2%)	275 (84.4%)	
Non-Hispanic Black	5 (3.8%)	0 (0%)	1 (1.4%)	0 (0%)	6 (1.8%)	
Hispanic	9 (6.9%)	9 (17.3%)	6 (8.1%)	2 (2.9%)	26 (8.0%)	
Other	8 (6.1%)	5 (9.6%)	4 (5.4%)	2 (2.9%)	19 (5.8%)	
Education *n* (%)						0.05
High school or less	7 (5.3%)	0 (0%)	3 (4.1%)	4 (5.8%)	14 (4.3%)	
Some college	22 (16.8%)	13 (25%)	8 (11%)	4 (5.8%)	47 (14.5%)	
College graduate or higher	102 (77.9%)	39 (75%)	62 (84.9%)	61 (88.4%)	264 (81.2%)	
Household Income ($) *n* (%)						0.43
Less than $29,999	6 (4.7%)	0 (0%)	1 (1.4%)	2 (2.9%)	9 (2.8%)	
$30,000–$49,999	14 (10.9%)	6 (11.8%)	7 (9.6%)	5 (7.2%)	32 (10.0%)	
$50,000–$69,999	16 (12.5%)	4 (7.8%)	15 (20.5%)	9 (13%)	44 (13.7%)	
At least $70,000	92 (71.9%)	41 (80.4%)	50 (68.5%)	53 (76.8%)	236 (73.5%)	
Body Mass Index *n* (%)						0.08
Less than 20	5 (3.8%)	0 (0%)	0 (0%)	1 (1.4%)	6 (1.8%)	
20 to 24.9	59 (44.4%)	22 (42.3%)	44 (59.5%)	34 (48.6%)	159 (48.3%)	
25 to 29.9	31 (23.3%)	15 (28.8%)	15 (20.3%)	24 (34.3%)	85 (25.8%)	
30 or greater	38 (28.6%)	15 (28.8%)	15 (20.3%)	11 (15.7%)	79 (24.0%)	
Previous Pregnancy Losses *n* (%)						0.71
No prior pregnancies	46 (35.1%)	19 (36.5%)	30 (40.5%)	33 (47.1%)	128 (39.1%)	
At least 1 prior loss	9 (6.9%)	4 (7.7%)	7 (9.5%)	5 (7.1%)	25 (7.6%)	
Prior Pregnancy, No Previous Losses	76 (58%)	29 (55.8%)	37 (50%)	32 (45.7%)	174 (53.2%)	
Smoking *n* (%)						0.59
None	121 (91%)	49 (94.2%)	70 (94.6%)	66 (94.3%)	306 (93.0%)	
Moderate (at least one day of 1–9 cigarettes per day)	8 (6%)	1 (1.9%)	2 (2.7%)	4 (5.7%)	15 (4.6%)	
Heavy (at least one day of 10 or more cigarettes per day)	4 (3%)	2 (3.8%)	2 (2.7%)	0 (0%)	8 (2.4%)	
Alcohol Consumption *n* (%)						0.18
Non-Drinker	46 (34.6%)	11 (21.2%)	26 (35.6%)	22 (31.4%)	105 (32.0%)	
Occasional Drinker (1–16 drinks per month)	72 (54.1%)	30 (57.7%)	31 (42.5%)	34 (48.6%)	167 (50.9%)	
Drinker (17 or more drinks per month)	15 (11.3%)	11 (21.2%)	16 (21.9%)	14 (20%)	56 (17.1%)	
Caffeine Intake *n* (%)						0.19
None	64 (48.1%)	23 (44.2%)	24 (32.9%)	24 (34.3%)	64 (48.1%)	
Moderate (1 cup per day)	48 (36.1%)	17 (32.7%)	32 (43.8%)	26 (37.1%)	48 (36.1%)	
Heavy (2 or more cups per day)	21 (15.8%)	12 (23.1%)	17 (23.3%)	20 (28.6%)	21 (15.8%)	

**Table 3:** Hazard ratio (HR) of loss in women becoming pregnant by iodine status (ratio <1 indicates reduced risk)—reference group—iodine sufficient women (*n* = 329).

Iodine Status ^a^	HR of Pregnancy Loss (95% CI) All Women Unadjusted	HR of Pregnancy Loss (95% CI) All Women Adjusted ^b^	HR of Pregnancy Loss (95% CI) No Hypo or Hyper-Thyroid Adjusted ^c^	HR of Pregnancy Loss (95% CI) No Hypo-Thyroid Adjusted ^c^	HR of Pregnancy Loss (95% CI) No Treated Hypo or Hyper-Thyroid Adjusted ^c^	HR of Pregnancy Loss (95% CI) No Treated Hypo-Thyroid Adjusted ^c^
Iodine—Continuous Variable (Log-Transformed)	1.11 (0.89, 1.38)	1.10 (0.81, 1.49)	1.08 (0.79, 1.47)	1.07 (0.78, 1.47)	1.09 (0.81, 1.47)	1.10 (0.82, 1.48)
Iodine Sufficient	Reference Group	Reference Group				
Severe Deficiency (<50 µg/L)	0.66 (0.37, 1.18)	0.69 (0.32, 1.50)	0.74 (0.34, 1.62)	0.69 (0.30, 1.59)	0.70 (0.30, 1.52)	0.67 (0.31, 1.45)
Moderate Deficiency (50–99 µg/L)	0.82 (0.48, 1.39)	0.81 (0.43, 1.51)	0.89 (0.47, 1.67)	0.95 (0.48, 1.90)	0.81 (0.44, 1.51)	0.81 (0.43, 1.51)
Mild Deficiency (100–149 µg/L)	0.65 (0.34, 1.24)	0.69 (0.34, 1.38)	0.67 (0.33, 1.39)	0.75 (0.36, 1.58)	0.69 (0.35, 1.39)	0.69 (0.34, 1.38)
Iodine Creatinine Ratio ^d^						
Iodine—Creatinine Ratio (Log-Transformed)—Continuous Variable	1.04 (0.80, 1.36)	1.05 (0.80, 1.38)	1.02 (0.77, 1.36)	1.01 (0.76, 1.35)	1.04 (0.74, 1.38)	1.05 (0.79, 1.38)
Severe Deficiency (<50 µg/L)	0.58 (0.18, 1.85)	0.53 (0.16, 1.76)	0.53 (0.16, 1.76)	0.53 (0.16, 1.76)	0.54 (0.16, 1.77)	0.53 (0.16, 1.74)
Moderate Deficiency (50–99 µg/L)	0.77 (0.43, 1.39)	0.76 (0.41, 1.40)	0.83 (0.45, 1.56)	0.82 (0.44, 1.53)	0.76 (0.41, 1.40)	0.76 (0.41, 1.41)
Mild Deficiency (100–149 µg/L)	0.85 (0.48, 1.50)	0.72 (0.38, 1.36)	0.80 (0.42, 1.51)	0.80 (0.42, 1.50)	0.72 (0.38, 1.36)	0.69 (0.37, 1.31)

^a^ Women in the underweight and normal weight groups were combined in the analysis because of the small numbers. ^b^ Adjusted models: adjusted for woman’s age, difference between the partner’s ages, woman’s race/ethnicity (referent: non-Hispanic white), woman’s education (referent: high school or less), household income (referent: less than $29,999), woman’s BMI (referent: underweight/normal), time-to-pregnancy, creatinine (log-transformed), average daily smoking in periconception window, average daily alcohol consumption in periconception window, diabetes (referent: non-diabetic) and previous losses (referent: not pregnant), history of hypothyroidism (referent: no), history of hyperthyroidism (referent: no). ^c^ Adjusted models: same as above except for history of hypothyroidism (referent: no), history of hyperthyroidism (referent: no). ^d^ Adjusted models: Same as above except for creatinine.

## Supplementary Materials

The following are available online at https://www.mdpi.com/2072-6643/11/3/534/s1, Spline function to assess non-linear association between iodine concentration and risk for pregnancy loss. Y axis indicates regression coefficient for risk for pregnancy loss. X axis indicates log transformed iodine concentration.
