# Supplementary figures and images for: Pregnancy Loss and Iodine Status: The LIFE Prospective Cohort Study
Source: Nutrients. 2019 Mar 1;11(3):534. doi: 10.3390/nu11030534 (PMC6471412; doi:10.3390/nu11030534)

Association between Iodine and Risk for Pregnancy Loss

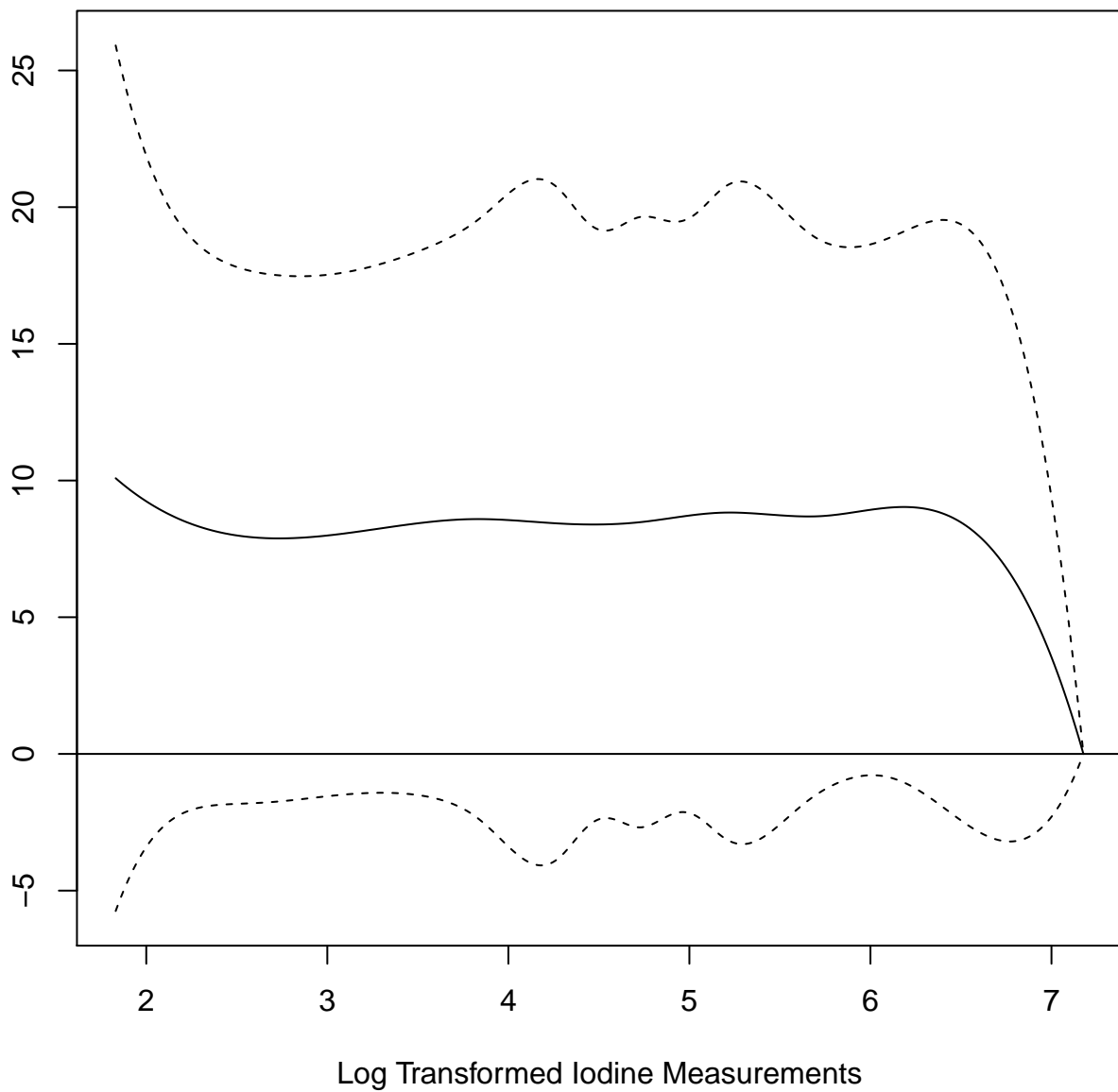

Supplement: Supplementary file 1 [file nutrients-11-00534-s001.pdf]
